# Using LTI Dynamics to Identify the Influential Nodes in a Network

**DOI:** 10.1371/journal.pone.0168514

**Published:** 2016-12-28

**Authors:** Goran Murić, Eduard Jorswieck, Christian Scheunert

**Affiliations:** 1 Communications Theory, Communications Laboratory, TU Dresden, Saxony, Germany; 2 Dresden Leibniz Graduate School, Leibniz Institute of Ecological Urban and Regional Development, Dresden, Saxony, Germany; Semmelweis University, HUNGARY

## Abstract

Networks are used for modeling numerous technical, social or biological systems. In order to better understand the system dynamics, it is a matter of great interest to identify the most important nodes within the network. For a large set of problems, whether it is the optimal use of available resources, spreading information efficiently or even protection from malicious attacks, the most important node is the most influential spreader, the one that is capable of propagating information in the shortest time to a large portion of the network. Here we propose the *Node Imposed Response (NiR)*, a measure which accurately evaluates node spreading power. It outperforms betweenness, degree, k-shell and h-index centrality in many cases and shows the similar accuracy to dynamics-sensitive centrality. We utilize the system-theoretic approach considering the network as a Linear Time-Invariant system. By observing the system response we can quantify the importance of each node. In addition, our study provides a robust tool set for various protective strategies.

## Introduction

A number of technical, social or economic systems are comprised of interconnected entities (computers, people, countries, etc.) [1]. Those interacting agents, affecting each other’s states are usually modeled as networks with nodes (vertices) representing the objects and links (edges) representing their relations. The nature and the amount of possible interactions and its effects make the number of available states of the system incredibly large, thus making the analysis of system dynamics computationally demanding [2]. Even for the relatively “predictable” behavior where agents could exist in only a few possible states, the system complexity exponentially grows with the number of agents involved. The behavior of less predictable agents, such as people, where the resulting state of the agent depends on various factors, makes the analysis of such systems especially challenging.

The complex interdependencies in various natural and man-made systems could be characterized as networks. For this reason network analysis became an important tool for studying some of the typical system dynamics such as spreading of information or diseases. Spreading characterizes numerous processes observed in social and communication networks [2], such as the spread of rumors, news and ideas among humans, or data broadcast and cyber attacks on communication networks. In order to control or prevent some of the spreading processes, it becomes imperative to understand the role of certain network elements. Therefore, identifying the most important nodes in regard to the spreading phenomena emerged as an important area of research [3–5].

There are various approaches for evaluating the node importance in the network. The most typical are based on *centrality measures* which quantify how much the node is centrally positioned regarding the network topology, such as degree, betweenness, closeness, local rank, h-index, Katz and eigenvector centrality [6–9]. Not any centrality measure could be used to identify important nodes for all spreading processes. In social networks, for identifying individual spreaders, k-shell is usually more reliable than degree [10]. Even though centrality measures efficiently recognize the most central nodes by identifying the hubs, they are not always efficient in capturing the spreading power of the vast majority of remaining peripheral nodes [11]. The sources of the infection are usually not the hubs, but rather the nodes which are not obviously influential. The centrality measures are not powerful enough to capture the influence of non-hubs, hence other metrics like *expected force* is introduced [12].

Besides the conventional centrality measures which assess the node’s importance based mostly on the path lengths and distances, another group of measures emerges which try to explain more specifically the *spreading power* or the *influence* of the node. The paramount objective is to determine the most important *spreaders* [5, 13]. The *k-shell* [14], *k-truss* [3], *percolation* [15], *accessibility* [16], *dynamic influence* [4], or *expected force* are used to identify the most important nodes able to spread the disease quickly through the network.

In this paper we propose such a measure, named *Node Imposed Response (NiR)*, which captures the node’s spreading potential. It can accurately classify the most important nodes demonstrating the high correlation with the simulation results. The measure outperforms *betweenness*, *degree*, *coreness* and *h-index* centrality in identifying the most influential spreaders in the case of the SI and SIR spreading processes. *NiR* does not depend on any parameters. However its performance is comparable even to the centrality measures which require variable parameters, such as *dynamic sensitive centrality (DS)* [17]. Proposed *NiR* measure utilizes concepts from system theory where the response of the system is evaluated for a certain number of inputs and outputs. We show that LTI approach could be used for the studying of spreading processes on networks thus uncovering the potential of the LTI modeling in network analysis. The proposed approach can be used to identify various potential relations from one or more sources to one or more end nodes only by manipulating inputs and observing different outputs in the corresponding LTI system.

First, we describe the principles of representing the network dynamic in linear time-invariant form. Then, we define the *NiR* and show the calculation steps using simple example. We validate *NiR*’s potential by simulating SI and SIR dynamics on real and generated networks. Finally, we discuss the results and give suggestions for future work.

### Notation

The main notations are listed and ordered as they appear in the paper.

*G*(*V*, *E*): graph/network consisted of set of vertices (*V*) and set of edges (*E*)
*N*: number of vertices (nodes) in the network
*M*: number of edges (links) in the network
*A*_*adj*_: adjacency matrix
*a*_*ij*_: weight of a link in the network. For the adjacency matrix, *a*_*ij*_ can be zero or one
$\underline{x}\left(n\right)$, $\underline{u}\left(n\right)$, $\underline{y}\left(n\right)$
state, input and output vectors respectively at discrete time *n*
*A* and *B*: state transition and input matrix which together define the properties of the system
*C* and *D*: output and feedforward matrix which are determined by the choice of output variables
1(n)
unit step function in discrete form
*H*(*s*): system transfer function
*y*(*t*): system response in the time domain
$L^{-1}$
inverse Laplace transform operator
*S*_*i*_: maximum value of step response with the single input in node *i*
*NiR*(*i*): node imposed response of the node *i*
*p*: spreading rate—the probability the node will infect a susceptible neighbor in one discrete time step
*μ*: recovery rate—the probability the infected node will recover in one discrete time step
*τ*: Kendall’s Tau coefficient
*E* [*X*(*p*)]: expected time of full infection with the probability of infection *p*
*S*_*max*_(*p*): maximum value of step response as a function of *p*

## Linear Time-Invariant Representation of Networks

Beside the most usual graph theoretic approach for dealing with nodes assessment, an alternative paradigm used here comes from the area of systems theory. The most prominent use of the system theory in dealing with problems related to the networks refers to the problem of controllability of dynamical systems [18]. The systems theory approach is used to identify the driver nodes necessary to control the system’s dynamics. The very nature of complex networks, consisting of multiple interconnected components communicating with each other, inspires the idea of conversion to the Multiple-Input and Multiple-Output (MIMO) system. In this paper we show that the systems theory approach can capture the most common epidemic dynamics: Susceptible-Infected (SI) and Susceptible-Infected-Recovered (SIR) models, regardless of the probability of infection.

A network is usually represented as a graph *G*(*V*, *E*), with *N* = |*V*| vertices or nodes and *M* = |*E*| edges or links. The network topology is usually characterized by the adjacency matrix *A*_*adj*_. Alternatively, the topology of the network could be identified by the edge list, meaning the *M* × *C* matrix where *M* is the number of edges and *C* is the information on edge. The minimal value of *C* equals 2 and usually *C* = 3 where the first, second, and third column are consisted of source node, sink node, and the edge weight respectively. The *N* × *N*, or adjacency matrix representation of the network topology, is more convenient in this case as it is easily transferred to the *A* matrix of the corresponding LTI system. For a graph with *n* nodes, *A*_*adj*_ is the *n* × *n* matrix where *a*_*ij*_ = 1 if the *i*^*th*^ and *j*^*th*^ nodes are connected, and *a*_*ij*_ = 0 otherwise. This particular graph representation is convenient for a system theoretic approach as it resembles the state matrix used in the state space representation of a physical system.

The nature of disease spreading is a transmission of certain unwanted information (i.e. virus) which enters the network at one or more points. It is subsequently replicated and conveyed from one node to another and the virus locations (or state of the network) change with every time step. The topology of the network is considered to be static. These properties allow us to observe the network as a discrete LTI MIMO (Linear Time-Invariant, Multiple Input—Multiple Output) system [19]. The state-space representation describes the system as:

$$\underline{x}\left(n+1\right)=A\underline{x}\left(n\right)+B\underline{u}\left(n\right)$$ (1)

$$\underline{y}\left(n\right)=C\underline{x}\left(n\right)+D\underline{u}\left(n\right),$$ (2)

where $\underline{x}\left(n\right)\in R^{N}$ is the state vector at discrete time *n*, $\underline{u}\left(n\right)\in R^{M}$ is input or control vector, and $\underline{y}\left(n\right)\in R^{M}$ is the output. The matrix $A:={\left(a_{ij}\right)}_{N\times N}\in R^{N\times N}$ is the state transition matrix and the matrix $B\in R^{N\times M}$ is input matrix. The matrix $C\in R^{M\times N}$ is the output matrix and $D\in R^{M\times M}$ is the feedforward matrix.

The matrix *A* determines the dynamics of the system and it can be obtained as a transpose of the adjacency matrix describing the network topology $A=A_{adj}^{T}$ [18, 19]. We can imagine the signal excites the system by entering one node. Then, the signal spreads over the network and each time it reaches the node it gets amplified by a certain parameter and subsequently transferred to all adjacent nodes. During this process, we can choose which nodes to observe, either all or just a fraction of nodes and measure the signal strength over time. We can interpret the measurement points as a set of sensors collecting data in every time step. By analyzing the gathered data, we can examine the dynamics in the network and estimate the possible impact of infecting certain nodes. The system matrices are generated the same way, regardless of the network dynamic we want to observe. Only the system response to the input signal is used for the analysis.

The LTI approach provides a tool for such a system analysis allowing us to gather and observe the signals from the set of designated nodes. We can choose to excite more than one node, hence simulating the multiple infection points.

**Fig 1:**
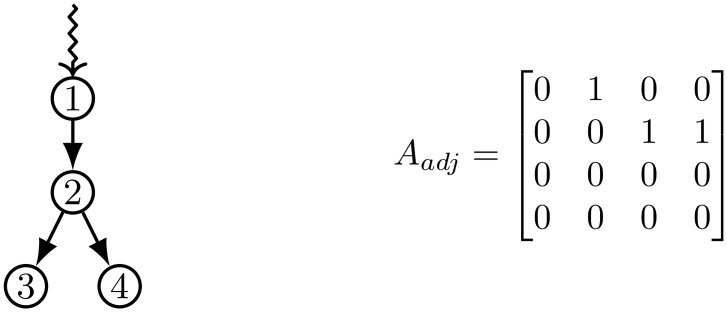
An example of a small directed network and its corresponding adjacency matrix. The zig-zag line shows the signal (virus) entering the network.

For example, a small directed network is shown in Fig 1 with the corresponding adjacency matrix *A*_*adj*_. The corresponding state-space representation of the system with matrices *A*, *B* and *C* is shown in Eqs (3) and (4). In this example the input vector $\underline{u}\left(n\right)$ is in the form of the Heaviside (unit) step function. Here we chose to observe all nodes, and therefore the matrix $C\in R^{M\times N}$ consists of all ones.

$$\underline{x}\left(n+1\right)= [\begin{array}{cccc} 0 & 0 & 0 & 0\\ 1 & 0 & 0 & 0\\ 0 & 1 & 0 & 0\\ 0 & 1 & 0 & 0 \end{array}]\;\underline{x}\left(n\right)+ [\begin{array}{c} 1\\ 0\\ 0\\ 0 \end{array}]\times 1\left(n\right)$$ (3)

$$\underline{y}\left(n\right)= [\begin{array}{c} 1\\ 1\\ 1\\ 1 \end{array}]\times \underline{x}\left(n\right)+ [\begin{array}{c} 0\\ 0\\ 0\\ 0 \end{array}]\times 1\left(n\right)$$ (4)

One can choose any preferred way for solving the system equations [20]. The system could be presented in the form of the transfer function *H*(*s*) where

$$H\left(s\right)=\frac{Y(s)}{X(s)}=C{\left(sI-A\right)}^{-1}B+D.$$ (5)

Furthermore, the step response of the system is obtained by

$$y\left(t\right)=L^{-1}\left(\frac{1}{s}H\left(s\right)\right)$$ (6)

However, this method could be challenging for large graphs as finding the inverse of large matrices is computationally expensive. An alternative and more programming-friendly approach would be a recursive solution [19] which avoids the matrix inversion. For a more detailed explanation refer to the example in the Discussion section.

## Calculating the NiR

*NiR* is the normalized maximum value of the step response *S*_*i*_ for the corresponding LTI system with the node *i* as the input. Let us define the maximum value of step response for the node *i* as *S*_*i*_, then

$$S_{i}=\max_{1<t<k}y_{i}\left(t\right)$$ (7)

The function *y*_*i*_, defined in Eq (6) is concave and eventually reaches its maximum value for a large enough *t*. Therefore *S*_*i*_ will always exist. Then

$$NiR\left(i\right)=\frac{S_{i}-S_{min}}{S_{max}-S_{min}},$$ (8)

where *S*_*max*_ = max_*j* ∈ {1, …, *n*}_ *S*_*j*_, *S*_*min*_ = min_*j* ∈ {1, …, *n*}_ *S*_*j*_, and *n* is the number of nodes in the network.

In order to calculate *S*_*i*_ we have to construct the corresponding LTI system which is defined by system matrices *A*, *B*, *C* and *D*. Using the transpose value of the adjacency matrix $A_{adj}^{T}$ we create the corresponding LTI system like in Eqs (3) and (4). In order to maintain the system’s bounded-input, bounded-output (BIBO) stability, the topology should be modified so the cycles are removed (see Discussion for details). The *NiR* could be calculated only for the acyclic directed graphs. An alternative version of *NiR* for graphs with cycles is discussed in the Discussion section. For the spreading processes, such as SI and SIR, cycles could be considered as irrelevant as the nodes cannot be infected twice. Also, removing the edges which form the cycles should not significantly influence the spreading dynamic. However, the algorithms for cycle removal do change the topology in the way some paths become excluded, especially for the undirected networks where one has to choose between the edge direction. Therefore, the proper way of removing cycles has to be chosen in order to maintain the most important paths from the source node and to introduce the minimal number of removed edges. Details about the method of producing acyclic graph are provided in Methods and Data section.

Next we create a system matrix *A* such that $A=A_{adj}^{T}$. All non-zero entries are substituted with the value *d* so ∀*a*_*ij*_ = 1: *a*_*ij*_ = *d*, and *d* < 1. Choosing *d* ≪ 1 is preferred. Supplementary investigation shows the variance of the *NiR* values for all nodes becomes higher as the value of *d* decreases. In the case of any relatively large network (*n* > 100) there is a large fraction of nodes with similar spreading power when considering the number of all non-hubs. In this case, the low variance could lead to false estimation, especially considering the removal of cycles and some of the topology information which gets lost during the process. Therefore, choosing the smaller *d* is important for proper node differentiation. In our simulations we use *d* = 0.1.

The step response of the obtained system will eventually reach the maximum value *S*_*max*_. That particular value calculated for the input node and normalized over all nodes within the range [0, 1] is the *NiR*.

### Small network example

**Fig 2:**
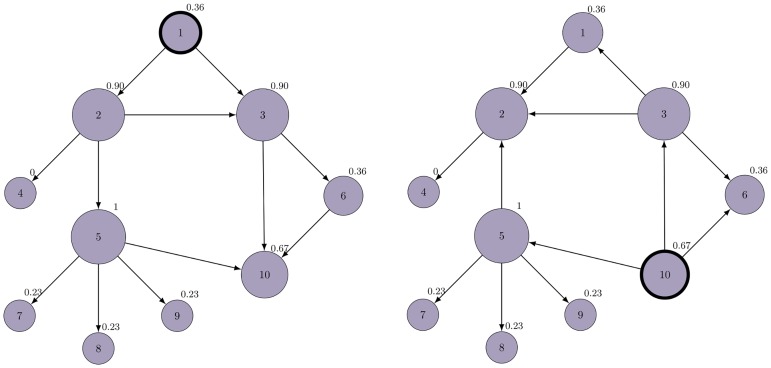
The *NiR* of all nodes in the network. For each node, we can calculate the *NiR*. Initially, the undirected network is made directed and acyclic with the respect to the source node. This is a necessary step in order to maintain the system’s stability. The LTI system is then formulated having in mind the new topology and the source. The value of the maximum step response of the corresponding system is the *NiR*. There are two topologies depicted for two observed nodes: node 1 (left) and node 10 (right). The normalized *NiR* values of all nodes are shown. The radius of the node represents the same (larger the radius, larger the *NiR*. Here we can identify the node 5 as the one with highest *NiR*.

In Fig 2 an example of a small network with *n* = 10 and corresponding *NiR* values is shown. In order to obtain *NiR*, the topology of the network has to be modified such that the cycles are eliminated. In this particular example we see two acyclic topologies: one for the source node ID1 (left), and second for the source node ID10 (right). The *NiR* value indicates the node’s spreading power, which means the node with higher *NiR* will infect the entire or the large fraction of the network faster.

**Fig 3:**
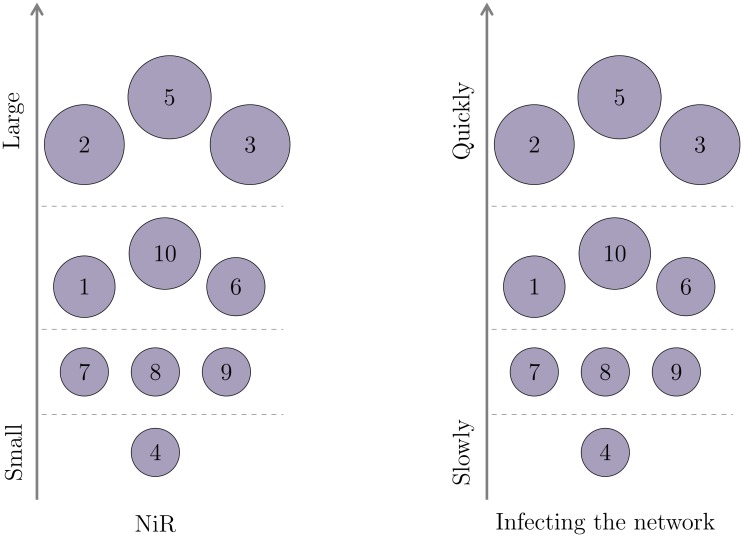
Nodes in the small network classified by the importance. On the left side, the nodes are ranked by their *NiR* value. Then, the SI infection is simulated for each source node and the time measured until the infection reaches all nodes. The right-hand picture shows the node ranking by the time infection needed to spread if the infection starts from that particular node. If the time for full infection is shorter, the node is ranked higher. The *NiR* measure could capture the node’s spreading power and put it in the right category. The distinct separation in groups by the spreading potential is made for the sake of the better visual presentation.

The claim is supported by simulating the SI spreading dynamic and comparing the results with the obtained *NiR* value for the small network which serves as an example (Fig 2). The infection starts from each node and the time the infection will reach all nodes is measured. If the time of full infection is shorter, the node has a potential to spread the infection faster and is considered more important (i.e. more influential). In order to compare *NiR* value and simulated spreading potential, we sort nodes by their *NiR* and spreading power obtained by the simulation (Fig 3). Based on those values the nodes are ordered and assigned to several distinct groups. This is the way we identify the nodes with high or low spreading potential. In the case of the example network, the *NiR* accurately captures the node’s spreading potential since the order of the nodes remains the same as if they were ordered by the time to the full infection. Normally for larger networks where *n* ≫ 10, there will be many nodes with similar *NiR*, hence the difference between consequent *NiR* values would not be as evident as in the example here.

## Simulation Results

**Fig 4:**
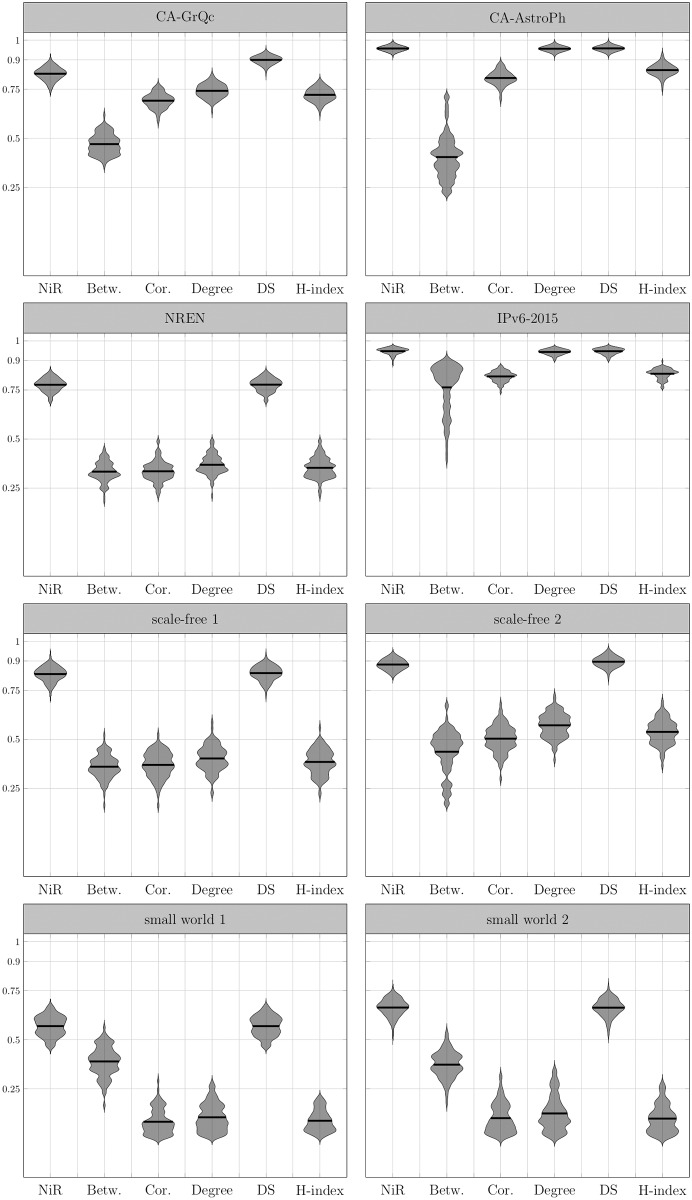
Correlation of *NiR* and centrality measures to the spreading outcome on simulated networks—the case of the SI model. Violin plots show the distribution of correlations between observed spreading dynamic and various centrality measures on 100 generated networks from each network family. Graphs are generated from the sample degree sequence of the real graphs. The correlation with *NiR* is relatively strong and outperforms betweenness, coreness, degree and H-index centrality with higher mean values and low variance. The vertical position of the violin plot demonstrates the correlation coefficient over all observed samples: the higher the position, the stronger the correlation. Additionally, the length of the plot indicates the variance: the more stretched plot, the bigger the variance. Therefore, the preferred plot is narrow and positioned high on the grid. The SI spreading process is used as a reference and it is computed as the time the infection reaches at least 50% of all nodes starting from the source node for the spreading rate of *p* = 0.05. The infection time is calculated as a mean time for 300 simulated processes for each observed node. The horizontal line within the plots shows the arithmetic mean of the correlation coefficients.

**Fig 5:**
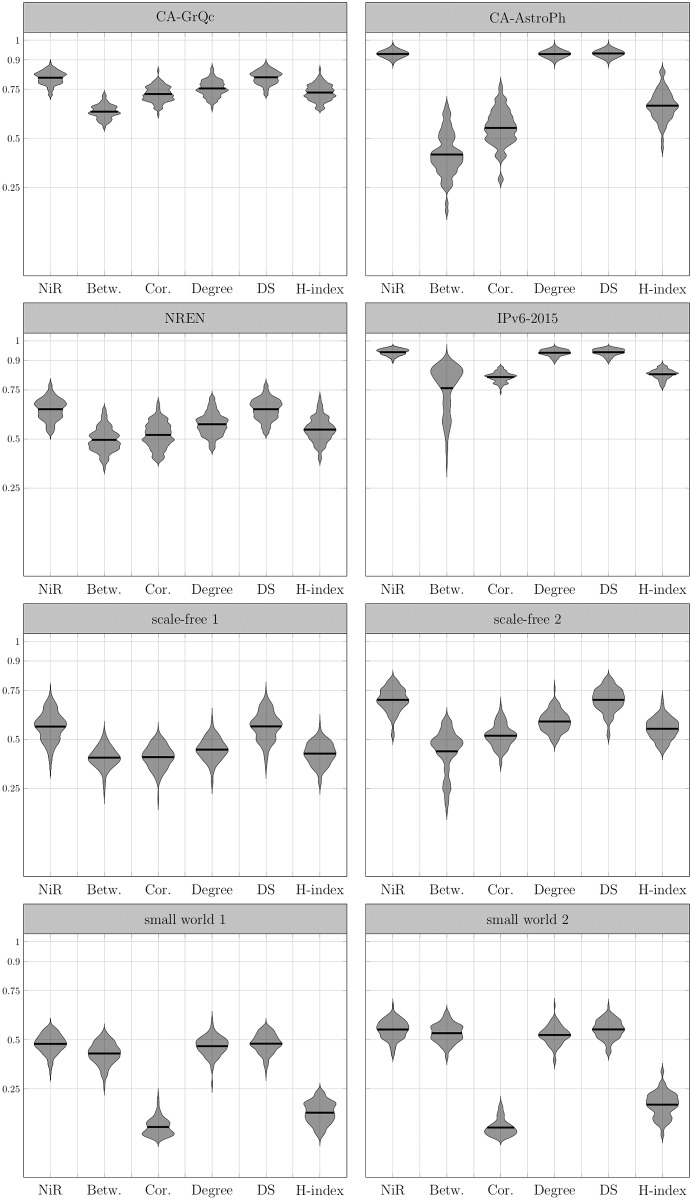
Correlation of *NiR* and centrality measures to the spreading outcome on simulated networks—the case of the SIR model. Violin plots show the distribution of correlations between observed spreading dynamic and various centrality measures on 100 generated networks from each network family. Graphs are generated from the sample degree sequence of the real graphs. The correlation with *NiR* is relatively strong and outperforms betweenness, coreness, degree and H-index centrality with higher mean values and low variance. The vertical position of the violin plot demonstrates the correlation coefficient over all observed samples: the higher the position, the stronger the correlation. Additionally, the length of the plot indicates the variance: the more stretched plot, the bigger the variance. Therefore, the preferred plot is narrow and positioned high on the grid. The SIR spreading process is used as a reference and the benchmark measure is the outbreak size of the infection when the spreading rate is *p* = 0.05 and recovery rate is *μ* = 1. The outbreak size is calculated as a mean outbreak size for 300 simulated processes for each observed node. The horizontal line within the plots shows the arithmetic mean of the correlation coefficients.

In order to confirm our assumptions, we first simulate the SI and SIR spreading processes on the set of graphs and compare it to the *NiR* measure. The results show a high correlation between the *NiR* measure and the outcome of the spreading processes in all families of networks that we used (Table 1). The correlation diagrams are shown in the form of the violin plots in the Figs 4 and 5.

**Table 1:** Generated and extracted networks. Four networks are generated using Barabási-Albert and Watts-Strogatz models for *scale-free* and *small-world* networks respectively. The rest are the real world networks of various sizes and characteristics taken from: *SNAP—Stanford Large Network Dataset Collection*, *UCLA’s Beyond BGP:Internet Topology Project* and *The Internet Topology Zoo*. All data sets are available online. Column *nodes* represents the number of nodes in the original network. Columns *diameter*, *density* and *clust. coeff.* represent the mean values calculated from the set of sampled networks. The *avg. degree* is the same for both the original and sampled networks.

*network*	*nodes*	*diameter*	*density*	*avg. degree*	*clust. coeff.*	*source*
scale-free 1	6000	14.69 ± 4.68	7.09*e*^−06^	3.40	0.0944	Generated
scale-free 2	6000	17.78 ± 10.22	8.75*e*^−07^	4.25	0.1148	Generated
small-world 1	6000	41.46 ± 31.54	3.95*e*^−06^	3.08	0.1602	Generated
small-world 2	6000	26.77 ± 16.23	4.99*e*^−07^	5.00	0.2070	Generated
CA-AstroPh	18772	9.54 ± 3.45	1.10*e*^−07^	21.25	0.2143	SNAP
ca-GrQc	5242	7.93 ± 4.50	4.17*e*^−07^	6.13	0.5296	SNAP
IPv6-2015	34761	5.19 ± 9.81	2.37*e*^−07^	10.54	0.0853	UCLA
NREN	1157	20.69 ± 7.31	2.97*e*^−06^	3.21	0.0994	Topology Zoo

We compare the simulation results against five other centrality measures (betweenness, coreness, degree, DS and H-index centrality). The *NiR* measure demonstrates high correlation to simulation results together with the low variance, often outperforming all five measures both in SI and SIR model. The only measure which performs equally is a DS centrality whose parameters depend on the dynamics. In the Figs 4 and 5, a single violin plot is displayed within the vertical and horizontal axes. The vertical axis represents the correlation between the experimental results and a specific measure. For the correlation value (ranging from -1 to 1) the width of the violin can be read. The violin width represents normalized correlation frequency obtained from multiple experiments. The higher the plot is positioned, the stronger correlation between measure and simulation results. Likewise, if the plot is positioned low, the correlation is weaker. The vertical length of the violin describes the robustness of the measure. If the plot is short, then the measure correlates with the simulation results most of the time with no large variations. The more vertically stretched plot demonstrates the higher variance in measures.

Note that all violin plots are smoothed for the sake of the better presentation. For smoothing, we estimate the probability density function of the observed correlation distribution using normal kernel density with kernel density estimate as *KDE* = 0.15 [21].

Not all nodes are used as sources for the measurements and comparison. In the case of the large networks, the incremental difference in the centralities or other measures between nodes is negligible. Therefore it is justified to choose a set of representatives in each group of nodes. Here we sort nodes based on their *NiR* value. Then, we divide the sorted set into 10 equal blocks. From each block we pick 8 nodes uniformly at random. In total, we choose the set of 80 nodes for each network to compare. This way the nodes are selected at random with regards to their importance, so the random number generator will not end up choosing too many similar nodes. Our assumption is that higher resolution would not add to the precision while significantly increasing the need for more computing power.

**Fig 6:**
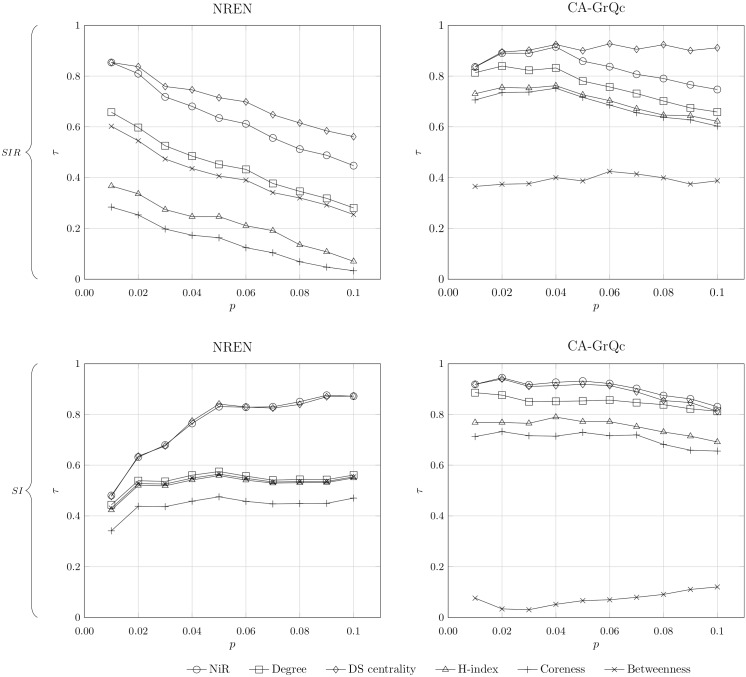
Correlation between centrality measures and spreading potential evaluation for various *p*. The probability of infection *p* takes a value from 0.01 to 0.1. Each data point is obtained by averaging over 10^4^ individual runs. Plots are generated according to the SIR model (upper half) and SI model (lower half). All correlations are quantified by the Kendall’s Tau coefficient *τ*.

Additionally, we simulate SI and SIR spreading processes on two real world networks (*NREN* and *CA-GrQc*) for various probabilities of infection *p*. As shown in Fig 6, *NiR* performs well in both networks for both spreading models, clearly outperforming degree, H-index, coreness, and betweenness centrality. For the SI model, *NiR* performs equally as well as DS centrality, even though it does not use any additional parameters from the spreading model. The centrality measures generally perform well in identifying the important spreaders for low infection probabilities. As the probability of infection increases, the accuracy of centrality measures slightly decreases, which means the identification of the influential spreaders becomes more difficult if the spreading rate is high.

## Methods and Data

### Spreading models

#### SI (Susceptible-Infected) epidemic model

In the SI model the node could be in one of two states: a) susceptible to infection and b) already infected and able to spread the infection. The epidemic process starts from the initially infected node. All neighboring nodes are considered susceptible. At each discrete time step the infected node attempts to infect all neighboring nodes independently with the probability of transmission *p*. For the healthy (uninfected) node *i* with *k* infected neighbors, the probability of infection in the next time step is *p*_*i*_ = 1 − (1 − *p*)^*k*^. The spreading process on the connected graph will eventually affect the whole network. We simulate the SI infection process which starts from the set of chosen nodes and measure the time until 50% of all nodes are infected. The time *t* needed for 50% infection is used as a basic benchmark. The results are similar if the infection threshold is raised to 70% or to the full infection. The *NiR* values for the same set of chosen nodes are then compared and the correlation is measured. This same process is repeated for 100 networks in each network family.

#### SIR (Susceptible-Infected-Recovered) epidemic model

In the SIR model, the node could take one of three states: a) susceptible to infection, b) already infected and ready to spread the infection and c) recovered (or removed) node that have been infected and will never be infected again. The epidemic process starts from a single infected node. All neighboring nodes are considered susceptible. At each discrete time step the infected node attempts to infect all neighboring nodes independently with the probability of transmission *p*. After that, each infected node recovers independently with the probability *μ*. For the healthy (uninfected) node *i* with *k* infected neighbors, the probability of infection in the next time step is *p*_*i*_ = 1 − (1 − *p*)^*k*^. At the same time the probability of recovery remains the same regardless of the node’s surroundings. Here we consider *μ* = 1, as the results should be very similar for other values of *μ* [17]. The benchmark value is an outbreak size (the number of infected nodes) after *t* time steps. Here, we limit the simulation time to *t* = 10.

### Networks

There are two types of networks used in the simulations. The first type are the *scale-free* and *small world* networks constructed randomly using various parameters (Table 1). Scale-free networks are constructed based on the Albert-Barabasi model of preferential attachment [22] using the algorithm described by Batagelj [23] and implemented in “A Controllable Test Matrix Toolbox for MATLAB” [24]. The first group of scale-free networks has a minimum node degree of 1, while the second group has a minimum node degree of 2. Those parameters affect the diameter and the density of networks, and therefore the expected dynamic of spreading processes. Similarly, the small-world networks are constructed with the same MATLAB tool using the Watts-Strogatz model [25]. The Watts-Strogatz model is based on two parameters which define the number of nearest neighbors to connect (*k*) and the probability of adding the shortcut in the given row (*p*_*s*_). The first group of generated small world networks has *k* = 1 and *p*_*s*_ = 0.5, while the second group has *k* = 2 and *p*_*s*_ = 0.5. The variety of initial parameters ensures the generation of networks with different properties such as *diameter*, *density* or *the average degree*. Randomly generated networks are connected, undirected and consisted of 6000 nodes each.

The networks in the second group are derived from the large real-world networks data. These real world networks are taken from various network dataset repositories: *SNAP Datasets: Stanford Large Network Dataset Collection* [26], *UCLA’s Beyond BGP:Internet Topology Project* [27], and *The Internet Topology Zoo* [28]. Two networks represent the collaboration pattern between authors of the papers submitted to arXiv; the *ca-GrQc* for the General Relativity and Quantum Cosmology category, and the *ca-AstroPh* for the Astro Physics category. The other two are technological networks which illustrate the topology of networked systems. The Internet AS-level topology network (*IPv6-2015*) is the monthly snapshot of AS-to-AS links as they appeared in the January 2015. The European network of National Research and Education Networks (*NREN*) is the backbone network managed by GÉANT which connects all European scientific and research institutions.

All simulations are conducted on network samples obtained from the available network sets. Sampled networks are characterized only by the degree distribution. Simulated networks are generated by 1000 node samples taken uniformly at random without repetition and by extracting the degree sequence. Networks are then constructed from the obtained degree sequence using the Havel-Hakimi algorithm [29]. Since the algorithm doesn’t guarantee the construction of connected graphs, the simulation is conducted on the largest connected component of the obtained network.

Graphs are usually not characterized only by the degree distribution. Constructing the graph from the degree sequence ignores some aspects of network topology such as communities. Because of the graphs sizes used here, the communities structures could not be generated in the same way they are represented in the original graphs [12]. Some other aspects of network characteristics such as costs, constraints, and direction on edges [30] could also be ignored as they are irrelevant in our undirected unweighted simulated networks. Network sampling process by extracting the degree distribution introduces a certain amount of randomness. To achieve the certainty of the correlation measurements, many network samples have to be generated. Each of the networks within the real world network family is generated over 100 times. For each of the random realizations of the topology, we measure the correlation of SI and SIR spreading dynamic and observed measures.

### Benchmark measures

The node’s importance is usually characterized by some of the numerous centrality measures. Centrality measures rank nodes by their potential influence. They are based mostly on the length of the walks which include the particular node. Some of them could also be parameterized [31].

**Degree** of a node is the number of edges (links) incident to the node [32]. In directed networks there is a difference between *indegree* *d*_*in*_(*i*), showing the number of immediate links directed towards the node *i* and *outdegree* *d*_*out*_(*i*) counting the number of links directing away from the node *i*. For the undirected networks there is one degree measure *d*(*i*) = *d*_*out*_(*i*) = *d*_*in*_(*i*). Degree is the simplest yet most robust measure of the node importance. Although it has certain drawbacks, especially in capturing the node importance in networks with many large clusters divided by the nodes with low degree, in most cases the degree accurately identifies the majority of the most influential nodes.

**Betweenness** represents the number of shortest paths from all nodes to all others that pass through a particular node. The value is usually normalized in the range [0, 1]. The betweenness centrality *b*_*k*_ of the node *k* is defined as follows [6]:

$$b_{k}=\sum_{i}\sum_{j}\frac{g_{ikj}}{g_{ij}}$$ (9)

where *g*_*ij*_ is the number of geodesic paths from node *i* to node *j*, and *g*_*ikj*_ is the number of geodesic paths from *i* to *j* that pass through *k*. There are some variations in the betweenness centrality based on the various approaches of defining the most desirable path. In some cases the constraints in the network make geodesic path not desirable, since it could be too costly (e.g. congested, expensive etc.). The actual betweenness centrality of the node is then modified taking in account also the weights of the links.

**Coreness** is the centrality measure derived from the k-core (also called k-shell) decomposition process of the network. The k-core is the largest subgraph comprising nodes of degree at least *k* [33]. A k-core of the graph can be obtained by recursively removing all the nodes of degree less than *k*, until all nodes in the remaining graph have at least degree *k*. The coreness *c*_*i*_ of a node *i* is *k* if the node belongs to the k-core but not to the (k + 1)-core [34]. By observing the coreness measure, we can identify the best individual spreaders in the network if the spreading process originates in a single node [13].

**H-index**, or Hirsch index, was originally used to measure the citation impact of the author. The H-index concept was later extended to quantify the importance of the node in the network. The H-index of a node is defined to be the maximum value *h* such that there exists at least *h* neighbors of degree no less than *h* [35]. It is interrelated to *coreness* and the *degree*, and it outperforms both measures in several cases.

**Dynamics sensitive centrality** integrates the topological features and the dynamical properties at the same time [17]. While the all other centrality measures used for comparison rely solely on the topological features, DS introduces two parameters, *β* and *μ*, representing the rate of the infection and the rate of the recovery respectively. The DS centrality is therefore particularly suitable for identifying the most influential spreaders when the SIR epidemic model is concerned. However, to properly assess the node’s importance one has to know the spreading dynamics parameters in advance.

Furthermore, all networks used in simulations are characterized by various global properties. Network attributes such as *diameter*, *density*, *average degree* and *clustering coefficient* are used to recognize the network model and to identify in which extend network topologies contrast to each other.

### Correlation measure

For all the analyses we use Kendall’s Tau rank correlation coefficient. It is a non-parametric measure of relationship between ranked data. The correlation coefficient *τ* takes a maximum value of 1 if the observations have identical rankings and a minimum value of -1 if observations have dissimilar rank. For each node *i* we calculate the spreading influence *x*_*i*_. In the case of SI model, *x*_*i*_ is as a time needed to infect the 50% of the network. For SIR model, *x*_*i*_ is the number of infected and recovered nodes. We calculate the *y*_*i*_ for each of the centrality measures (i.e. *NiR, betweenness, coreness, degree, H-index, DS*) for the node *i*. Nodes are then ranked using $\bar{x}$ and $\bar{y}$, and the rankings are compared. The accuracy of the observed measure is compared to the simulated spreading dynamics using Kendall’s Tau [36] as:

$$\tau =\frac{2}{n(n-1)}\sum_{i<j}sgn\left[\left(x_{i}-x_{j}\right)\left(y_{i}-y_{j}\right)\right],\;sqn\left(y\right)=\left\{\begin{array}{cc} 1, & y>0\\ -1, & y<0\\ 0, & y=0 \end{array}\right.$$

### Obtaining acyclic graph

To calculate the maximum value of the step response and therefore the *NiR* the system by definition has to be BIBO stable. The number of cycles in the original graph will make the system unstable for any value of *a*_*ij*_ within the *A* system matrix. Thus, the topology of the observed graph has to be modified so the cycles are removed and at the same time the number of nodes remains unchanged. In the process of modifying the topology, the number of removed edges should be minimized in order to maintain the topology as similar as possible to the original. One has to consider keeping the most critical (or most probable) paths for infection spreading, such as shortest-path tree with the source node as the parent. Furthermore, in the process of edge removal, the edges closer to the source node should be given priority since the importance of the topology decreases quickly with the distance from the seed [12, 31, 37]. The procedure used here for making the graph acyclic is as follows: *(1)* identify the source node; *(2)* extract the shortest paths tree (SPT) with the source node as the parent; *(3)* direct the SPT edges away from the source node; *(4)* return excluded edges iteratively starting from the edges closest to the source; *(5)* if the returned edge forms the cycle, remove it.

## Discussion

The proposed *NiR* metric can successfully predict the epidemic dynamic in various network models. The results of the numerical simulations show a relatively large correlation with the actual infection process. This metric also shows a small variance, which demonstrates the high robustness regardless of the type of network. The underlying paradigm based on the LTI system approach allows for the numerous variations of the metric. By choosing the proper nodes as inputs and outputs, one can use the similar approach for solving various other problems. For example, we can identify the nodes which are more likely to be reached from the set of other nodes by observing the output at the given nodes. The weighted networks can be further analyzed using the same approach by including the weight values in the *A* matrix.

In order to evaluate the spreading power of the node, the most important area of interest is its surrounding. The importance of topological information decays quickly with the distance from the observed node. Therefore, almost all centrality measures could rely with high confidence on local neighborhood information [12, 38]. The *NiR* shows a similar property. The signal strength decreases with each time step as it is being amplified with the factor of *a*_*ij*_ ≪ 1. Even though multiple incoming edges boost the signal strength by summation, each node decreases the resulting signal with the parameter *a*_*ij*_ at the same time. Take for example the line graph consisting of *n* nodes connected consecutively. If the resulting system gets excited by the unit impulse signal at the first node, the signal strength after *t* time steps will be already 10 × *t* times weaker for the *a*_*ij*_ = 0.1. This implies the signal strength observed in nodes which are 4 steps away from the source is already highly attenuated. The observations made on any nodes positioned even further from the source could be irrelevant for the *NiR* assessment. This property makes it possible to evaluate the node importance with *NiR* using just the knowledge of the local topology, thus allowing the measure to be effectively used for very large graphs.

**Fig 7:**
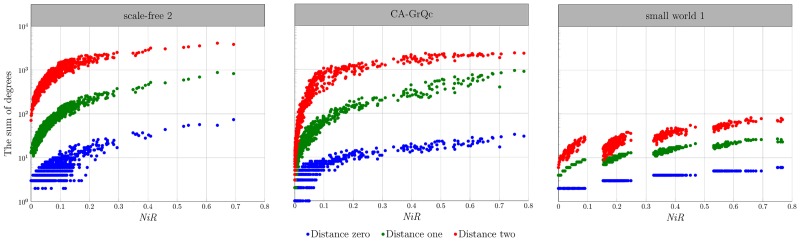
The correlation between degree and the *NiR*. There is a strong correlation between degree and *NiR* value for all types of the networks observed. The correlation against the node degree for all networks takes a value of 0.94 ± 0.02. The sum of all degrees of the node’s neighbors (degree of the distance one) correlates even more with the *NiR* value when the correlation coefficient is 0.98 ± 0.01. Random scale-free network, as well as the General Relativity collaboration network (*ca-GrQc*) show the expected pattern on the graph clearly demonstrating the presence of the small number of hubs, compared to the relatively large number of non-central nodes. On the other hand, the small-world model generates approximately the same number of nodes which could be grouped by the importance.

In the case of all equal edge weights, the *NiR* shows high correlation to node degree at the distance *k* (Fig 7). The interesting observation is that the correlation with the node degree at distance one (0.98 ± 0.01) is higher than with the degree of distance zero (0.94 ± 0.02). Both *NiR* and the degree at the distance one cover the relevant area around the node more broadly than the simple degree. It can also be noted that the topology information loss when calculating the *NiR* for the immediate proximity of the source node is negligible. As we go further away from the source node, the correlation between *NiR* and the degree decreases. For the distance two, correlation already drops to 0.94 ± 0.1. The *NiR* still exhibits the higher overall correlation compared to the infection dynamics, which demonstrates the *NiR’s* ability to incorporate more topological information than a degree.

The main motivation behind limiting the analysis solely to the acyclic graphs is the BIBO system stability. A system is BIBO stable if there is a bounded output for every bounded input over the time interval [*t*_0_, ∞). Since the *NiR* is defined as a normalized *maximum* value *S*_*max*_ of the step response, the system has to have bounded output. To obtain the BIBO stability without adjusting the link weights, the cycles have to be removed. For the stable system, the amplitudes of the system response could be observed and analyzed at any desired time step, while the response of the unstable system quickly reaches extremely high absolute value. The systems observed here are not real physical systems, but rather their mathematical model. For the sake of the measurement we can allow the system to be unstable and let cycles exist. In that case, we have to read the response quickly after the initial excitement. Our preliminary results show that the measure derived in such a way exhibits the same or even better performance than the proposed *NiR*. The analysis of the unstable systems will be the topic of our further research.

In future work the additional information regarding the networks can be considered. The values in adjacency matrix and consequently in the *A* system matrix can be unequal, therefore allowing the various costs of the links. Furthermore, an additional constraint such as edge directions could be introduced.

### Reasoning behind

**Fig 8:**
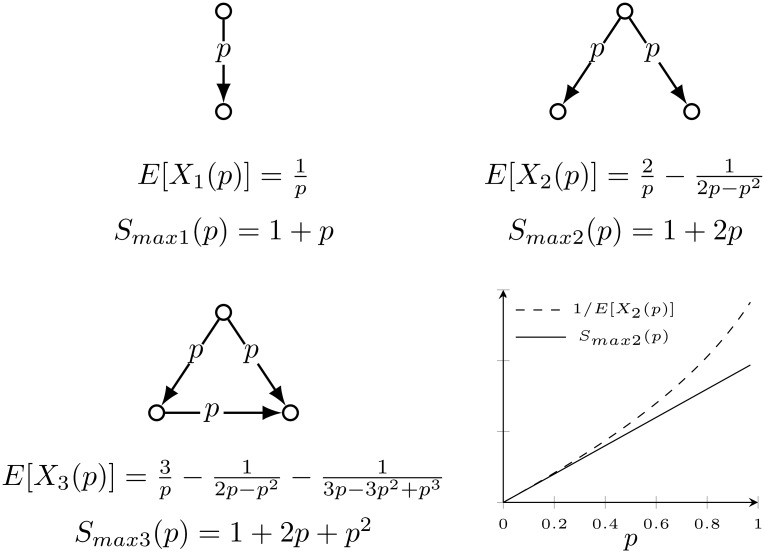
Expected time of infection and step response: small networks example. For three small networks the expected time of full infection, *E* [*X*(*p*)], is calculated. For all networks the source of the infection is the parent node (top). At each time step the parent node tries to infect neighboring susceptible nodes with the probability *p*. All nodes will be eventually infected and the time of full infection is presented with a certain distribution (i.e. the distribution of the expected number of trials in discrete time for the infection to reach all nodes). The *E* [*X*(*p*)] is the mean of the distribution for each network (the expected number of trials before the success.) The *S*_*max*_(*p*) is the maximum step response value of the corresponding LTI system.

**Fig 9:**
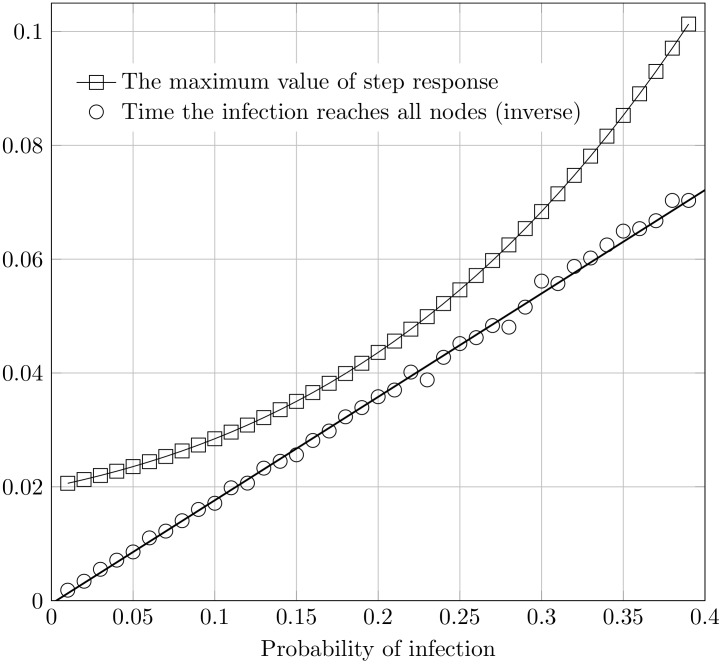
The comparison of the step response and the infection time. Here we compare the time the infection reaches all nodes and the maximum value of step response of the corresponding LTI system for the various probabilities of infection. For the sample random acyclic graph with 100 nodes, the corresponding single input LTI system is constructed. The non-zero values in *A* matrix range from 0.01 to 0.4, to capture the system behavior with various infection probabilities. The system is excited by step function and the maximum value of the step response is presented with data points as squares. The results indicate the exponential trend. On the other hand, we simulate the SI infection dynamic starting from the same node. The simulation is conducted for 40 different probabilities of infection (0.01–0.4). The time for the infection to reach all nodes is then measured (i.e. the time when the network becomes fully infected). For comparison, the inverse value is plotted with data points as circles and the black line exhibits the linear trend. Both curves are monotonically increasing.

The example of three simple graphs given in Fig 8 demonstrates that the value of the step response could be used to predict the simple spreading dynamic. We further argue that the same principle could be used for arbitrary directed acyclic graph of any size which we show in numerical simulation later (Fig 9). The main hypothesis underlying the claim is that the response value of the corresponding LTI system correlates with the time needed to infect the network or the large part of it.

It is intuitively known that the time delay between the initial infection and the complete infection is a function of the probability of virus transmission between the infected and susceptible node. This relation is reciprocal. The expected time all nodes will be infected *E* [*X*(*p*)] is a monotonic function for all nodes and therefore it could be used for the node’s ordering. On the other hand, the maximum value of the step response of the corresponding LTI system *S*_*max*_(*p*), presented as a function of *p* is monotonic as well, although increasing. The corresponding system is derived from the acyclic directed graph with non-negative values of the *A* matrix elements in the range 0 < *a*_*ij*_ < 1. The example of three small networks (Fig 8) and their corresponding *E* [*X*(*p*)] and *S*_*max*_(*p*) demonstrate this trend.

The illustration of the phenomenon becomes clear in this small example. Calculating the *E* [*X*(*p*)] for slightly larger graphs already becomes too complex.

To derive the expected time of infection for larger non-regular networks rapidly becomes too difficult as the number of nodes increases. However, the simulation results on those networks suggest the same monotonically increasing trend (Fig 9). The time the infection will spread rises monotonically with the increased probability of infection *p*, which is expected. At the same time, the maximum value of the step response follows the similar trend. This leads to the conclusion that those two measures (*expected time of the infection* and *maximum step response*) could be used interchangeably, except that *S*_*max*_ is considerably easier and faster to compute.

### Simple LTI infection pattern on a sample tree graph

**Fig 10:**
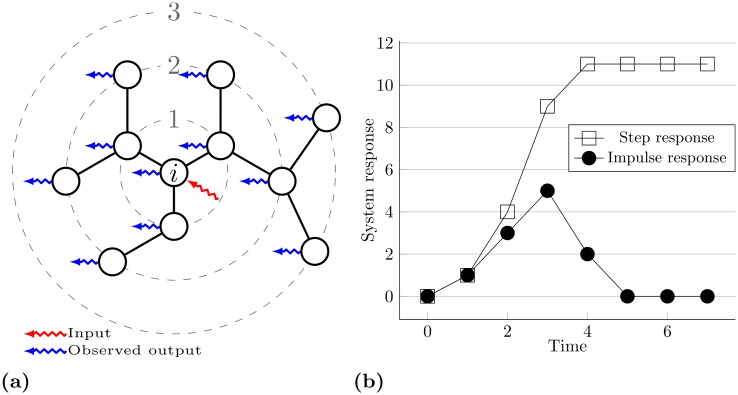
Simple graph and corresponding system response. (a) An example of a small tree graph where the node *i* is a source of the infection. The signal in the form of the unit step or impulse function enters the network at node *i*. Here we observe the state of the system in each time step by measuring the signal strength in all nodes and adding them together. The resulting measurements are step and impulse response respectively. (b) Step and impulse response of a corresponding LTI system with the single input in the position of node *i*. The response corresponds to the spreading dynamics over time which originates in the source node *i*. This is the simple case of almost certain infection of the neighboring nodes in each time step over the tree graph. The step response reveals the number of infected nodes over time. Impulse response shows the number of infected nodes in each time step.

Another more illustrative example of a small tree graph is presented in Fig 10(a). For the sake of clarity nodes are positioned on dotted circles each representing the hop distance from the parent node. The corresponding system matrices are created and the step and impulse response are calculated. Notice that matrix *A* still has ones as non-zero elements. Parent node *i* was chosen as an input (i.e. the source of infection). We observe all nodes as outputs and calculate the final output as the sum of signal strengths in all nodes over time. The sum output is plotted in Fig 10(b). Notice the *impulse response* for the unit input. The value of the response over time equals the number of nodes on corresponding circles. For the case of a virus transmission in the example network with the source in the node *i*, and the almost certain virus transmission from infected to susceptible (*p* = 1), the impulse response shows exactly the number of infected nodes over time. Likewise, the *step response* displays the total number of infected nodes.

This method for infection analysis is limited to tree graphs and assuming that the infection transmission from infected to susceptible is almost sure. An alternative method to overcome this limitation is network expansion with a specified number of intermediate nodes between each couple [19]. The contagion spreading is a process which is usually non-desired. Therefore, it is not usually characterized by the almost sure transmission rate. Moreover, the probability of infection is relatively small, ranging significantly below 100% (*p* ≪ 0.1). For the unlikely case of *p* = 1, the network could be transformed to a shortest path tree with the seed node since the parent as the infection route is known and unnecessary edges could be removed without affecting the infection dynamic. On the other hand, for any *p* < 1, the number of multiple incoming edges and multiple possible paths must not be neglected. Here we show that modification of the A matrix could lead to the LTI model of a system which can predict the infection dynamics on an arbitrary topology for any *p* < 1.
